# The Impacts of Discrimination and Filial Caregivers’ Age on Aspects of Physical Health

**DOI:** 10.1093/geroni/igaa057.1159

**Published:** 2020-12-16

**Authors:** Barbara Hodgdon, Jen Wong

**Affiliations:** The Ohio State University, Columbus, Ohio, United States

## Abstract

Filial caregivers (e.g., individuals caring for a parent or parent-in-law) are a part of the growing number of family caregivers in midlife and late adulthood. The responsibilities that filial caregivers navigate in midlife and late adulthood may expose them to multiple types of discrimination that may decrease their physical health, though this relationship has been understudied. As numbers of family caregivers grow, it is important to examine the potential vulnerability of younger and older filial caregivers’ physical health in the context of discrimination. Informed by the life course perspective, this study compares the physical health of younger (aged 34-64) and older (aged 64-74) filial caregivers who experience discrimination. Filial caregivers (N=270; Mage=53; SD=9.37) from the Midlife in the United States (MIDUS-II) Survey reported on demographics, family caregiving, daily discrimination, self-rated physical health, and chronic conditions via questionnaires and phone interviews. Regression analyses showed no differences between younger and older adults’ self-rated physical health or average chronic conditions. However, moderation analyses revealed that younger filial caregivers who experienced greater discrimination reported poorer self-rated physical health than their older counter parts as well as younger and older filial caregivers who experienced less discrimination. Additionally, younger caregivers with greater discrimination exposure exhibited more number of chronic conditions as compared to other caregivers. The study results highlight the impact of the intersection between filial caregivers’ age and discrimination on physical health. Findings have the potential to inform programs that could promote the health of filial caregivers in the face of discrimination.

